# Supplementary figures and images for: Utilizing random regression models for genomic prediction of a longitudinal trait derived from high‐throughput phenotyping
Source: Plant Direct. 2018 Sep 10;2(9):e00080. doi: 10.1002/pld3.80 (PMC6508851; doi:10.1002/pld3.80)

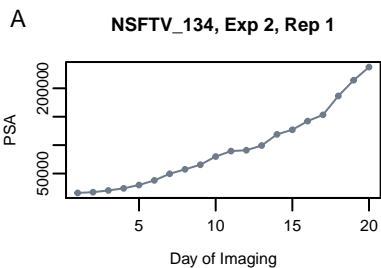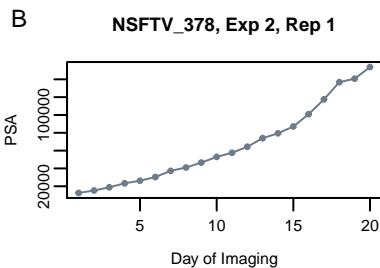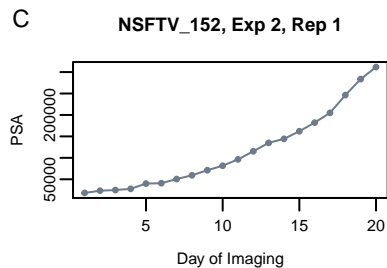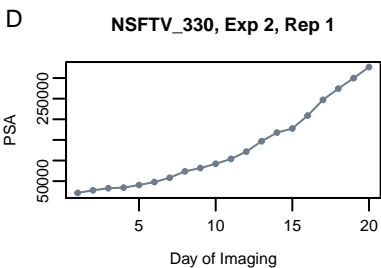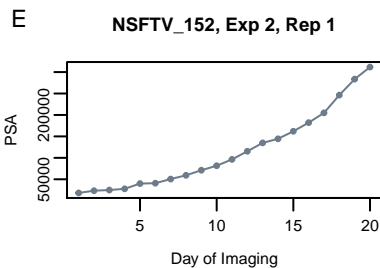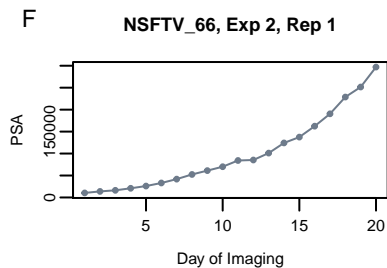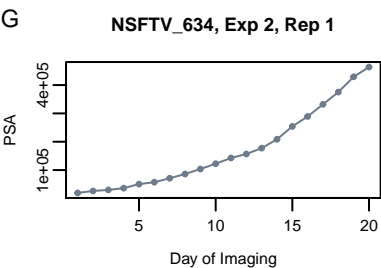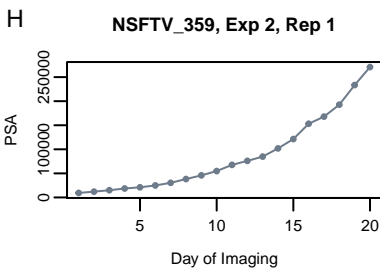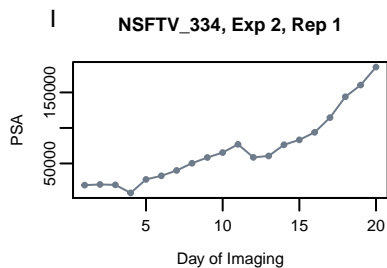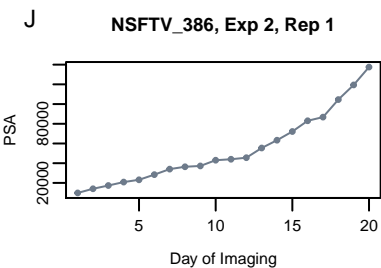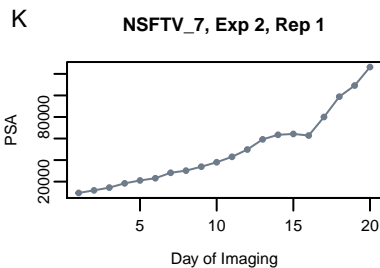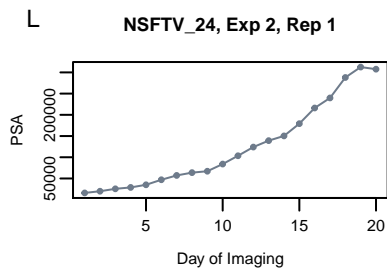

Supplement: Supplementary file 1 [file PLD3-2-e00080-s001.pdf]

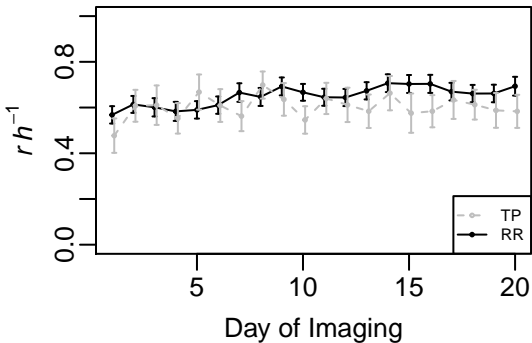

Supplement: Supplementary file 2 [file PLD3-2-e00080-s002.pdf]
