# Supplementary material for: Utilizing random regression models for genomic prediction of a longitudinal trait derived from high‐throughput phenotyping
Source: Plant Direct. 2018 Sep 10;2(9):e00080. doi: 10.1002/pld3.80 (PMC6508851; doi:10.1002/pld3.80)
Supplement: Supplementary file 3 [file PLD3-2-e00080-s003.docx]

# Supplemental Data

**Table S1. Random regression model selection.** Each of the four random regression models included a fixed second-order polynomial to model the mean trend in PSA over the twenty time points, indicated by the column $f$. $G$ refers to the random additive genetic effect, $Exp$ the random experimental effect, and $e$ error term. Models with $Diag$ assumed heterogeneous residual variance over time points, while those with $I$ assumed the residual variance was constant. $pol^{n}$ refers to a Legendre polynomial of order $n$.

| $Model$ | $f$ | $G$ | $Exp$ | $e$ | $LogREML$ | $AIC$ | $BIC$ |
| --- | --- | --- | --- | --- | --- | --- | --- |
| Model 1 | $pol^{2}$ | $pol^{0}$ | $pol^{0}$ | $I$ | 2026.97 | -4047.93 | -4023.65 |
| Model 2 | $pol^{2}$ | $pol^{0}$ | $pol^{0}$ | $Diag$ | 19358.83 | -38673.65 | -38495.60 |
| Model 3 | $pol^{2}$ | $pol^{1}$ | $pol^{0}$ | $I$ | 7345.85 | -14681.69 | -14641.23 |
| Model 4 | $pol^{2}$ | $pol^{1}$ | $pol^{0}$ | $Diag$ | 23273.62 | -46499.24 | -46305.01 |
| Model 5 | $pol^{2}$ | $pol^{2}$ | $pol^{0}$ | $I$ | 8204.64 | -16393.28 | -16328.54 |
| Model 6 | $pol^{2}$ | $pol^{2}$ | $pol^{0}$ | $Diag$ | 24718.93 | -49383.86 | -49165.35 |
| Model 7 | $pol^{2}$ | $pol^{2}$ | $pol^{0}$ | $I$ | 12700.64 | -25381.28 | -25300.35 |
| Model 8 | $pol^{2}$ | $pol^{2}$ | $pol^{1}$ | $Diag$ | 27537.59 | -55017.19 | -54782.49 |

Figure S1:Projected shoot area for a subset of 12 lines. The line identifier (NSFTV_), experiment (Exp), and replicate (Rep) are provided in the plot titles.

Figure S2: Predictive ability of the random regression (RR) and single time point (TP) approaches expressed as a function of heritability: The analysis followed the same approach as that for scenario A, however, for each fold the correlation between gBLUP and observed PSA was divided by the square root of heritability. The error bars represent the standard deviation where = 20.
