# Supplementary material for: Utilizing random regression models for genomic prediction of a longitudinal trait derived from high‐throughput phenotyping
Source: Plant Direct. 2018 Sep 10;2(9):e00080. doi: 10.1002/pld3.80 (PMC6508851; doi:10.1002/pld3.80)
Supplement: Supplementary file 4 [file PLD3-2-e00080-s004.pdf]

Reviewer #1:

This study described the use of random regression models to handle repeated HTP of plant growth. I feel that this is a timely study that touches two important topics: high throughput phenotyping and genomic prediction. Although authors seemed to focus more on modeling and analysis part, I think the value of these modeling can certainly guide us to better design HTP time point and better understand the physiology and genetics. Showing how RR can model this longitudinal data itself should be interesting to may plant biologists.

On the genomic prediction side, author attempted multiple scenarios and the simulation analysis was done well.

- Please come up with a diagram to show the model and parameters (final selected one) so readers can better understand the RR. This may be done by working on the Figure 2. Using the selected model from Table 1. Move the Table 1 into a supplementary table as this is not the focus of the study. You picked a model, which should be straightforward, then the follow-up analysis is the key.
  Response: In the revised MS, Table I has been moved to the supplemental data. We have also added a second panel to Figure 2 that shows the predicted PSA for two contrasting lines. In the figure legend, the gBLUPs for the RR coefficients are provided for each line.
- On capturing h2, I would assume that any analysis approaches that consider multiple time points would be better than TP. This should not be unique to RR?
  Response: Yes, this is expected. Since the correlation is very high between time points, it's likely that any approach that captures the covariance between time points (e.g. multivariate) would improve heritability estimates compared to a single time point approach.
- Figure 1 should be better organized.
  Response: We have edited Figure 1 so that all plots are aligned, and have clearly labeled the two datasets used for scenario D.
- Please provide a supplementary figure to show a random set of PSAs so readers can have a better understanding of the data.
  Response: A subset of 12 lines, from Experiment 1 are provided as Supplemental Figure S1.
- Table 1, Exp not "E" the random experimental effect.
  Response: The caption has been corrected. "*E*" now reads "*Exp*".
- L194. Typo for Sigma_g_2
  Response: Corrected.
- Discussion related to population structure is not needed, but okay to keep it. I suspect this was there due to an incorrect suggestion/comment. In the case of genomic prediction, there is nothing wrong with leveraging the embedded population structure, which itself is caused by genetics. Only when detecting individual genetic signal should we consider controlling for population structure.
  Response: The rice diversity panel consists of two subspecies, *Indica* and *Japonica*, and thus it is highly structured. In the manuscript we didn't mention any stratification within our germplasm, however we included the discussion about population structure to let the reader know that we are aware of the deep population structure in RDP1, but it is not important for the analyses performed in our study.
- I must have skipped the reason for having h-1 for Fig. 6. I would not worry about the closeness of RR and TP if h-1 was dropped from the y-axis. I think it is understandable that the difference in r between RR and TP may not be significant given the robust data collected across a diverse set of materials. But there will be cases when a smaller sample is used when TP fails to work well. Not sure whether authors want to explore this by subsampling, but this is not required.
  Response: We decided to express the prediction accuracy as a function of the square root of heritability to support our hypothesis that the greater prediction accuracy of the RR was due to the greater h2. While we acknowledge that subsampling would be an interesting approach, we feel that it is outside the scope of the current manuscript.

Reviewer #2:

Campbell et al. have produced a high quality manuscript. I would note only a few minor errors and one request for clarification:

- Lines 155-156 States that variations for u, s, and e were assumed to be normally distributed. Were they actually normally distributed?

Response: In our models, *u*, *s*, and *e* are considered as random effects. We assume that in our data the observed levels for these random variables are drawn from a larger population. Since we can't know the true distribution of random effects, we simply assume these effects follow a normal distribution with a mean of 0 and a variance as specified in the text. Since the original wording may have been misinterpreted we have clarified this in the revised text.

- Line 246 "fitted3"

  Response: Corrected.

- Lines 351-352 "The prediction accuracies for scenario B were very similar to those observed for scenario A" - do you mean C and A?

  Response: This sentence has been corrected and now reads “The prediction accuracies for scenario C were very similar to those observed for scenario A.”

- Line 363 "panel1"

  Response: This panel1 is in reference to Rice Diversity Panel 1. In the revised MS the name has been capitalized and reads “Rice Diversity Panel 1”.
